# Qualitative assessment in the third month of life allows for a better prognosis of the achievement of motor milestones versus assessment of pathological reflexes- prospective studies on Polish children

**DOI:** 10.3389/fpubh.2023.1253137

**Published:** 2023-09-14

**Authors:** Joanna Surowińska, Magdalena Sobieska, Ewa Gajewska

**Affiliations:** ^1^Manus Medica, Warsaw, Poland; ^2^Department of Rehabilitation and Physiotherapy, Poznan University of Medical Sciences, Poznan, Poland; ^3^Chair and Clinic of the Developmental Neurology, Poznan University of Medical Sciences, Poznan, Poland

**Keywords:** reflexes, qualitative assessment, motor performance, crawling, sitting down, walking

## Abstract

**Introduction:**

The characteristic feature of primitive reflexes is that they occur early in development and must expire at a well-defined age. The study was conducted prospectively on a group of 107 children (74 boys). The study population included 83 infants born on time (weight 3,465 ± 395 g) and 24 born prematurely (weight 2,225 ± 793 g).

**Methods:**

An analysis of motor development at 3 months of age consisting of a qualitative assessment (motor performance) and a check of reflexes was performed; at 9 months, the child was checked for crawling and sitting down, and at 16 months for walking.

**Results:**

The more abnormal reflexes, the less likely it was to achieve the assessed milestones in time. It is possible to notice that the qualitative assessment is, in each case, a better predictor of the milestones than any of the reflexes, except for walking, where the Babkin reflex was strongly predictive. However, the qualitative assessment sum still occupies the second and third positions in prone and supine positions.

**Discussion:**

The occurrence of Babkin’s reflex at 3 months of age impacts the achievement of sitting down and walking functions. An abnormal Galant reflex was strongly associated with the lack of occurrence of crawling on time. At the same time, a high-quality score at 3 months of age guarantees the development of crawling on time, sitting down, and walking.

## Introduction

The study of primitive reflexes and their evolution is one of the components of the neurodevelopmental evaluation of children. Vojta, the founder of analysis and neurodevelopmental therapy, intended the study of primitive reflexes to serve the early diagnosis of cerebral palsy. Other authors also undertook studies on reflexes and various neurological disorders in subsequent years (1–5). However, there is a lack of data on the relationship between the presence or absence of primitive reflexes and the achievement of milestones such as crawling, sitting down, or walking, including in children who are not at risk for significant deficits but show minor motor developmental disorders (2, 3, 6).

The characteristic feature of primitive reflexes is that they occur during early development and expire at a strictly defined age. The study of primitive reflexes should occur under appropriate conditions when the child is not drowsy, as this can distort the result of the study (1). In terms of developmental diagnosis, the study of primitive reflexes is an essential component for determining abnormalities in motor development (1).

Both the grasping reflex of the hand and the grasping reflex of the foot are very primitive, meaning they can be induced in all healthy premature infants as early as 25 weeks of fetal life. The grasp reflex of the foot is triggered by examining the child by pressing the thumb over the 2–3 heads of the metatarsal on the sole side. The correct reaction triggered is flexion of the toes at the metatarsophalangeal joints. Physiologically, the grasp reflex of the foot occurs until the child gains supporting function on the lower limbs (7).

The hand grasp reflex is used in diagnosing newborns and infants from birth and has applications in diagnosing cerebral palsy, for example. This reflex appears developmentally intrauterine from 16 weeks and can be triggered at birth very early, as early as 25 weeks in premature infants (8). The hand grasp reflex is triggered in the child in the supine position. The examiner presses on the baby’s hand from the inside with the examiner’s index finger inserted from the elbow side. In the case of the hand grasp reflex, the most vigorous response is observed up to the third month of life, with attention to the highest intensity up to the fourth week (7). The response is triggered in both upper limbs until the age of 6 months. The correct extinction of the hand grasp reflex is associated with the appearance of hand support and grasping (6). On the other hand, the development of normal support functions is used to achieve milestones such as crawling and sitting down (9).

The absence of the reflex in the first months of life, a more intense reaction after the third month, or the possibility of triggering it after the sixth month of life is considered a pathological pattern (1). The reaction one should expect after 3 months is finger closure on the examiner’s index finger, which consists of two phases: finger closure and holding. An abnormal reaction at this time is considered finger closure and clenching with the intensity that occurs in an infant up to 4 weeks of age or a complete lack of response to an attempt to trigger the reflex (3, 8).

The crossed extensor reflex is strongest in the first 6 weeks of an infant’s life and disappears by the third month of life. It is induced in the supine position by the examiner performing a maneuver of maximum flexion and simultaneous internal rotation at the hip joint and flexion at the knee joint. The reaction is evident on the free lower limb; that is, there is an extension of the free lower limb with simultaneous adduction and internal rotation at the hip joint and a horsefoot position. The abnormal reaction at 3 months is a persistent upright reaction in the free lower limb (3).

The suprapubic reflex occurs from birth to 3 months of age. The examiner triggers the reflex in the tested child, also in the supine position, by applying pressure to the infant’s pubic conjunctiva. At 3 months of age, the correct response is that the lower limbs do not react; they remain loosely placed on the ground. The abnormal response is a sudden straightening of the lower limbs, with simultaneous internal rotation and adduction at the hip joints, sole flexion of the foot and extension of the toes (3, 6).

The Galant reflex occurs from birth to 4 months of age; the person examining the child triggers it when the child is freely held in a position on the stomach upon the hand of the examiner. The stimulus is pressure with a finger or neurological hammer of the paravertebral region from the lower end of the scapula at the height of the 7–8th thoracic vertebra to the height of the 2–3rd lumbar vertebra. The correct response is the appearance of a concavity on the stimulated side. In contrast, the abnormal response at 3 months is a complete lack of response or a robust response involving the whole body, with an intensity like that of a newborn. The Galant reflex disappears after the fourth month of life (2, 3).

The Babkin’s reflex occurs from birth to 6 weeks of age, with the highest intensity by week 4. The examiner triggers it in the supine position by pressing firmly with the thumbs on both palms of the infant’s hands on the palm side of the hand. The correct response observed by week 6 is flexion of the head and trunk toward the chest with simultaneous opening of the mouth and closing of the eyes may occur. Obtaining Babkin’s reaction at a later age is considered a pathological reaction (6).

Over the past four decades, two things have become clear: 1- motor behavior is not organized primarily in reflex terms, and 2- as early as fetal age, the cerebral cortex modulates them (10). In particular, motor behavior is based on spontaneous, patterned activity, the quintessential function of neural tissue (10, 11). Such motor activity can be measured quantitatively and qualitatively.

As a qualitative assessment, we chose the third month of life considering that this is the base of future normal motor development (12–14); it should be emphasized that the age of 3 months after birth - or instead, the period from 2 to 4 months - is the age of significant changes in motor development (10). These consist of a transition from endogenously generated diverse movements that primarily explore and sculpt the nervous system to movements that can be increasingly modified and adapted to the constraints of the environment.

Motor development is characterized by variations in how tasks are accomplished (based on the available repertoire) and intra- and inter-individual variations in the rate at which milestones are reached. As a result, the age at which motor milestones are reached is highly dispersed - including across cultures (10).

However, for our study, as a result of literature analysis, we assumed that most healthy children achieve the function of independent sitting down and crawling around 9 months of age, social walking around 16 months of age according to Vojta’s neurodevelopmental diagnosis (14) and WHO studies (15).

Crawling, in our research, means moving around on open palms and knees. The torso is then raised above the ground owing to the straightened upper limbs, while the lower limbs are bent at the hip and knee joint; we observe reciprocal arm and leg movement with trunk rotation (16).

The term “walking pattern” means achieving bipedal locomotion on two lower limbs independently and freely. A child can move on straight lower legs, crossing a particular space, often called social walking (17, 18).

## Aim of the study

Comparison of qualitative motor assessment according to the “Quantitative and qualitative assessment sheet” with both the occurrence of individual reflexes and the number of pathological ones at 3 months and their influence on the achievement of selected milestones on time.

## Materials and methods

### Study group characteristics

The study included children receiving care from the Rehabilitation Clinic whose parents/guardians requested screening to exclude or confirm developmental abnormalities. Children referred for functional evaluation by pediatricians or neurologists were also included. The research was conducted between 2018 and 2020.

Demographic data on the child’s health status were taken from the health booklet or information sheets. The study was conducted prospectively on a group of 107 children, 33 girls and 74 boys. The study population included 83 children born on time, with an average weight of 3,465 ± 395 grams, and 24 infants born prematurely (but not below 28 gestation age), with an average weight of 2,225 ± 793 grams. Babies born prematurely were studied at the corrected age (19).

As to the type of delivery, 56 children were born by natural force, 49 by cesarean, and two were delivered through vacuum two children. Ninety-eight subjects were born in good condition according to Apgar scale scores (Apgar score between 7 and 10), 7 in medium condition (Apgar score 4–6) and 2 in severe condition (Apgar score < 4). Respiratory distress syndrome occurred in 9 children, while hypotrophy was found in 23 subjects; 23 had hyperbilirubinemia.

An evaluation at 3 months of age consisting of a qualitative motor performance assessment was performed (Panel A), and the foot grasping reflex, hand grasping reflex, crossed extension, suprapubic, Galant, and Babkin’s reflexes were checked. (Panel B) The child was checked for crawling and sitting down at 6 months and 16 months for walking (quantitative assessment).

#### Panel A

The qualitative assessment included 15 elements in the prone and 15 in the supine position.

In the prone position, the assessment involved: isolated head rotation; arm in front; forearm in an intermediate position; elbow outside of the line of the shoulder; palm loosely open; thumb outside, spine segmentally in extension; scapula situated in medial position; pelvis in an intermediate position; lower limbs situated loosely on the substrate; foot in an intermediate position. In the supine position, the assessment involved: head symmetry; spine in extension; shoulder in a balance between external and internal rotation; wrist in an intermediate position; thumb outside; palm in an intermediate position; pelvis extended; lower limb situated in moderate external rotation and lower limb bent at the right angle at hip and knee joints, foot in intermediate position – lifting above the substrate. Both sides were assessed for symmetrical parts of the body to exclude asymmetry. Each element was assessed as 0—performed only partially or entirely incorrectly, 1—performed correctly. Each assessed element had to be observed at least three to four times during the test. The result was expressed as a sum of points (0–15 for the prone and 0–15 for the supine position). Interobserver reliability ranged from 0.870 to 1.000, while intraobserver reliability was equal to 1 (9, 12, 13, 20). Previously, this type of examination was used in the assessment of children aged 3 months, and the comparison between physiotherapeutic and neurological assessment showed high agreement., with high conformity coefficients (*z* = −5.72483, *p* < 0.001) (20).

#### Panel B

Reflexes were checked as described in the introduction and classified as proper/improper for each reflex. In addition, the number of abnormal reflexes was summed for each child.

### Quantitative assessment at the age in the ninth month of life

All children were tested to determine whether they had achieved independent crawling and sitting down at 9 months of age. It was noted whether the child had reached a milestone (YES/NO). The child had to independently demonstrate the crawling function by covering a distance of at least 1 meter, sitting down independently and maintaining this position for at least 5 s.

### Qualitative assessment in the 16th month of life

The so-called social walking was considered during the evaluation at 16 months of age. It was noted whether the child had reached a milestone (YES/NO).

Walking was assessed by achieving an upright position (at the ladders/chair or independently), and then the child had to walk from one room to the next.

A physiotherapist performed the tests with many years of experience in performing functional diagnosis of children and a certified teacher of the Vojta method.

### Statistics methods

A description using the mean with the standard deviation was applied for interval variables with a normal distribution. Due to the nature of other variables, the results were presented as medians with quartiles (Me, Q25-Q75) and analyzed by non-parametric tests. Comparisons between groups by category and the achieved final level were performed using the Mann–Whitney U test for two groups or the Kruskal-Wallis ANOVA when more groups were compared (quality in the prone position, quality in the lying position). The assumed statistical significance level was *p* < 0.05. The validity of the predictors was assessed by regression; Statistica.pl. program was used.

## Results

The group was analyzed only for abnormal reflexes and qualitative assessment at 3 months of age, and no children were divided based on prematurity or the presence of risk factors. Children were divided according to the presence or absence of particular pathological reflexes, and for each division, the sum of the prone and supine position scores was counted. The presence of abnormal reflexes was associated with a lower qualitative score, and the difference was in each case statistically significant. Detailed data is presented in Table 1.

**Table 1:** The incidence of proper and improper reflexes versus qualitative assessment in prone and supine positions, in infants aged 3 months.

Reflex	Proper/improper, number of children; % of the whole group = 107	The sum in the prone position	The difference between children with proper/improper reflex	The sum in the supine position	The difference between children with proper/improper reflex
Foot grasp	Proper, *n* = 94; 88%	6 (2–10)	4.23; <0.001	7 (3–11)	4.44; <0.001
	Improper, *n* = 13; 12%	0 (0–1)		0 (0–1)	
Hand grasp	Proper, *n* = 60; 56%	10 (7–12)	5.94; <0.001	8 (6–12)	6.33; <0.001
	Improper, *n* = 47; 44%	2 (0–4)		2 (0–5)	
Crossed extension	Proper, *n* = 79; 74%	7 (4–10)	5.52; <0.001	8 (5–11)	5.77; <0.001
	Improper, *n* = 28; 26%	1 (0–3)		2 (0–4)	
Suprapubic	Proper, *n* = 74; 69%	8 (4–10)	5.85; <0.001	9 (5–11)	6.04; <0.001
	Improper, *n* = 33; 31%	1 (0–3)		2 (0–5)	
Galant	Proper, *n* = 37; 34%	10 (8–14)	6.56; <0.001	11 (9–13)	6.57; <0.001
	Improper, *n* = 70; 66%	3 (0–6)		4 (1–7)	
Babkin	Proper, *n* = 62; 58%	8 (6–12)	6.51; <0.001	10 (7–12)	6.39; <0.001
	Improper, *n* = 45; 42%	1 (0–4)		2 (0–5)	

The difference between the groups was assessed by U Mann–Whitney test, *z*-, and *p-*values are given.

The analysis of proper/improper reflexes in the examined children at 3 months of age revealed the most common abnormalities concerning Galant, Babkin, and hand grasp reflexes (Table 1). Next, children were classified according to the number of pathological reflexes they presented. The qualitative assessment (expressed as the sum in the prone and supine positions) was calculated for such division. The results showed decreasing motor performance and an increasing number of pathological reflexes, and the correlation between these variables was statistically significant.

**Figure 1:**
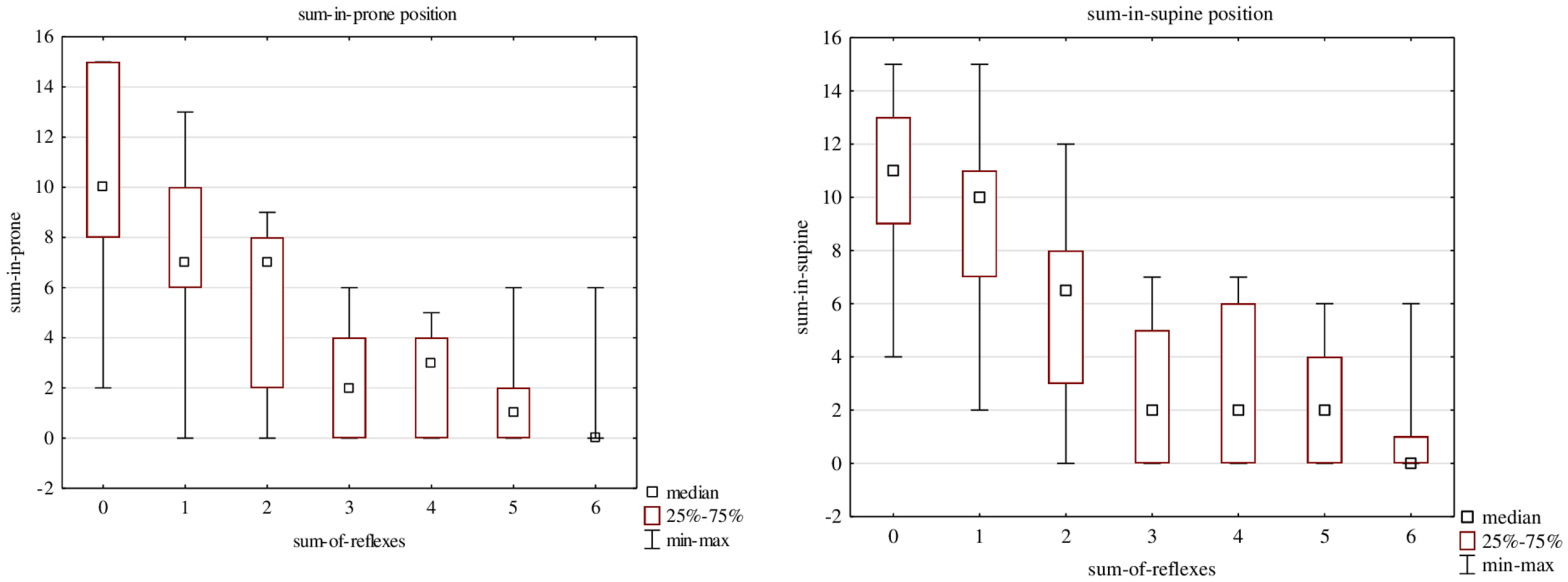
The quality of assessment in the prone and in the supine positions in Children who showed one to six improper reflexes.

When children were divided according to the number of improper reflexes they presented, the qualitative assessment decreased with the number of improper reflexes (Spearmann’s rho for the prone position −0.778 and the supine position −0.760, *p* < 0.05). The difference between the groups according to the number of improper reflexes was assessed using Kruskal-Wallis ANOVA; it was statistically significant (*H* = 67.06; *p* = 0.000) and is shown in Figure 1 for prone and supine positions. For the prone position, the following statistically significant differences were found with a *post-hoc* Dunn’s test: between the group without improper reflexes (0) and children with three (*p* = 0.000), four (*p*-0.004), five (0.000), or six improper reflexes (*p*-0.000).

For the supine position, the following statistically significant differences were found: between the group without improper reflexes (0) and children with two (*p* = 0.028), three (*p* = 0.000), four (*p*-0.001), five (0.000) or six improper reflexes (*p*-0.000) (Figure 1).

Next, children were classified according to the assessment of achieving the chosen milestones (crawling, sitting down and walking) on time. For each division, the number of pathological reflexes children showed when aged 3 months and the qualitative assessment (expressed as the sum in the prone and the supine positions) was calculated. It was proven that the more abnormal reflexes, the lower the probability of reaching typical milestones on time. At the same time, it was shown that the quality assessment at 3 months of life was significantly higher in children who reached milestones on time. The results are shown in Table 2.

**Table 2:** The number of improper reflexes and achieving the selected milestones on time: crawling, sitting down, and walking.

Milestone	Achieved versus not achieved on time	The sum of improper reflexes	The difference between achieved on time versus not achieved on time
Crawling	On time, *n* = 53; 50%	0 (0–1)	7.55; <0.001
	Not achieved on time, *n* = 54; 50%	4 (2–5)	
Sitting down	On time, n = 58; 54%	0 (0–1)	7.62; <0.001
	Not achieved on time, *n* = 49; 46%	4 (3–5)	
Walking	On time, *n* = 91; 85%	1 (0–3)	5.50; <0.001
	Not achieved on time, *n* = 16, 15%	5 (5–6)	

The difference was assessed using the Mann–Whitney U test; *z*- and *p-*values are given.

Finally, it was investigated which assessment system (the qualitative assessment in the prone and supine positions or the presence of pathological reflexes) better predicted achieving selected milestones. The validity of the predictors for the milestones assessed was calculated to evaluate it.

The importance of predictors was estimated for the three investigated milestones: crawling, sitting down, and walking. Each milestone was correct if achieved on time (crawling and sitting down at the latest 9 months, walking at the latest 16 months). The investigated reflexes and the qualitative assessment at 3 months in prone and supine positions were used for prognosis. The results are presented in Table 3. For each milestone, the three most important predictors are marked in bold. The qualitative assessment is, in each case, a better predictor of the milestones than any of the reflexes, except for walking, where the Babkin reflex is strongly predictive.

**Table 3:** The importance of predictors (qualitative assessment and pathological reflexes) for the three investigated milestones.

Predictor	Crawling	Sitting down	Walking
Sum-in-supine-position	**1.000**	**1.000**	**0.896**
Sum-in-prone-position	**0.984**	**0.907**	**0.810**
Galant	**0.865**	0.613	0.192
Babkin	0.783	**0.687**	**1.000**
Hand grasp	0.497	0.654	0.758
Crossed extension	0.496	0.479	0.810
Suprapubic	0.496	0.451	0.785
Foot grasp	0.205	0.286	0.689

The three most important predictors for each milestone are marked in bold.

Crawling and sitting down, assessed in the same month, also show a similar pattern in predictor validity. Babkin’s reflex proved to be the most predictive for the milestone of walking. However, the qualitative assessments still occupy the second and third positions: the sum in the prone and supine positions.

## Discussion

In our study, it was shown that children who crawled, sat down, and walked on time (crawling and sitting down up to 9 months were considered normal, and walking up to 16 months) had very high scores in the sum of elements in supine and prone positions, according to the validated sheet, confirming previous results (9, 12, 13).

The foot grasp reflex is a primitive reflex included in the diagnosis of the Vojta method and used during the standard neurological examination. Futagi wrote that asymmetry in the foot grasp reflex response indicates damage in the central nervous system. Futagi and Vojta recommend treating asymmetry in the foot grasp reflex response as a red flag suggesting neurological conditions (6, 21). No studies were found linking the foot grasp reflex directly to the observation of milestones. In our study, the foot-gripping reflex was the weakest predictor for sitting down and crawling and quite significant for walking.

The grasping hand reflex is also part of the diagnosis and is a critical reflex suggesting development toward spastic or dyskinetic forms of cerebral palsy, as presented by many authors (10). Chinello et al. showed that the hand grasp reflex is persistent in children with a future diagnosis of autism spectrum disorder (22), confirming the need for research on reflexes in children.

Our study showed that the hand grasp reflex had a weak relationship with the onset of crawling about the time and a slightly more substantial relationship with sitting down and walking. An abnormal hand grip reflex was associated with a significant reduction in the quality of motor development in prone and supine positions (see Table 1).

Persisted supinated and crossed pronation reflex reactions are a red flag to the researcher and indicate the development of a spastic form of cerebral palsy (6). The study’s authors showed that normal reflex responses are critical for achieving walking function in children with an extremely low qualitative assessment of motor development (defined as the sum in the supine and prone positions at the age of 3 months). A similar relationship was noted when analyzing the crossed upright reflex.

The very intense Galant reflex response, or lack thereof, described by Vojta and occurring at 3 months of age, is considered a pathological pattern (6). Schulze describes that at 3 months, only the trunk flexion reaction on the side of the triggered reflex remains without co-motions in the limbs. In our study, it was noted that the isolated abnormal response of the Galant reflex influences the abnormal time to reach milestones. Galant’s reflex had a decisive effect on the achievement of regular crawling on time, and its pathological occurrence at 3 months of age significantly reduced the quality of motor development.

Our study showed that infants having an abnormal Babkin’s reflex had significantly lower sum in the prone and supine positions than children with a normal response. The occurrence of this reflex still at 3 months of age resulted in children not achieving sitting down and especially walking about time. The study’s authors agree with Vojta’s view that at 3 months of age, the Babkin’s reflex is pathological and indicative of central nervous coordination disorders (6).

Reflex assessment is an essential component of testing in children whose motor development is poor on qualitative assessment. It can help identify children in need of therapy who cannot reach milestones due to neurological dysfunction. Primitive reflexes are not a significant predictor for diagnosing delayed crawling, sitting down, and walking functions. Infants with typical results of primitive reflexes show a much higher quality of motor development when compared to children with abnormal results. Therefore, the evaluation of motor development at 3 months of age should begin with a qualitative assessment in prone and supine positions. Decreased quality should imply more advanced assessment; among other tests, the evaluation of reflexes should follow.

The occurrence of a single pathological reflex was not associated with a significantly lower quality of motor assessment, while the more pathological reflexes, the lower the quality of motor development examined at 3 months of age, and these two totals correlated significantly negatively with each other. It seems that reflexes manifest more profound dysfunction on the highest floor of the motor system, and their occurrence should always be a warning sign. However, even in the absence of pathological reflexes in children whose quality assessed at 3 months of age was low, motor deficits should be reckoned with, surrounded by careful observation, and, if possible, given adapted therapy right away. Our previous observations also drew the same conclusion (9, 13, 20, 23).

### Strengths and limitations

We confirm statistical limitations due to the number of cases. Qualitative analysis was performed in the third month of life, regarded as a crucial time point to predict further development, allowing to plan therapy or social support in cases of expected disability. A relatively short follow-up (up to 16 months) is the limitation of the study.

## Conclusion

Qualitative assessment at 3 months has a high predictive value regarding achieving crawling, sitting down and walking on time. Particular attention should be paid to children in whom pathological Babkin’s and Galant’s reflexes were observed in the third month, as this may predict delayed achieving of milestones.

## Data availability statement

The raw data supporting the conclusions of this article will be made available by the authors, without undue reservation.

## Ethics statement

This study was carried out with the consent of the Senate Bioethics Committee Poznan University of Medical Sciences (1105/18). The studies were conducted in accordance with the local legislation and institutional requirements. Written informed consent for participation in this study was provided by the participants’ legal guardians/next of kin.

## Author contributions

JS, EG, and MS: conceptualization, formal analysis, writing—original draft, and writing—review and editing. JS and EG: data curation. EG: funding acquisition and project administration. EG and MS: investigation and resources. MS: methodology. All authors have read and agreed to the published version of the manuscript.

## Conflict of interest

The authors declare that the research was conducted in the absence of any commercial or financial relationships that could be construed as a potential conflict of interest.

## Publisher’s note

All claims expressed in this article are solely those of the authors and do not necessarily represent those of their affiliated organizations, or those of the publisher, the editors and the reviewers. Any product that may be evaluated in this article, or claim that may be made by its manufacturer, is not guaranteed or endorsed by the publisher.
